# *Pantoea ananatis*, A New Bacterial Pathogen Affecting Wheat Plants (*Triticum* L.) in Poland

**DOI:** 10.3390/pathogens9121079

**Published:** 2020-12-21

**Authors:** Krzysztof Krawczyk, Beata Wielkopolan, Aleksandra Obrępalska-Stęplowska

**Affiliations:** 1 Department of Molecular Biology and Biotechnology, Institute of Plant Protection—National Research Institute, 20 Węgorka St., 60-318 Poznań, Poland; k.krawczyk@iorpib.poznan.pl; 2 Department of Monitoring and Signalling of Agrophages, Institute of Plant Protection—National Research Institute, 20 Węgorka St., 60-318 Poznań, Poland; b.wielkopolan@iorpib.poznan.pl

**Keywords:** *Pantoea ananatis*, wheat, plant-pathogenic bacteria, cereal leaf beetle, wheat disease

## Abstract

Wheat (*Triticum aestivum*) is one of the most economically important crops in the world. During the routine monitoring of wheat pest, the cereal leaf beetle (CLB, *Oulema melanopus,* Coleoptera, Chrysomelidae), in the Greater Poland region, it was observed that some leaves wounded by CLB also displayed brownish lesions with clear margins and yellow halo, disease symptoms resembling a bacterial infection. The aim of this study was therefore to investigate those symptoms to establish a causal agent of the disease. The identification based on the results of the Biolog’s Gen III system, 16S rRNA, and *gyr*B genes sequencing, revealed the presence of eight strains of *Pantoea ananatis* bacteria. Four strains were derived from wheat leaves (Ta024, Ta027, Ta030, Ta046), and four from the CLB’s oral secretion (OUC1, OUD2, OUF2, and OUG1). They shared the nucleotide identity ranging from 99 to 100% to *P. ananatis* strains deposited in the GenBank database. Additionally, the multi-locus sequence analysis (MLSA) of concatenated sequences of partial *atp*D, *fus*A, *gyr*B, *rpl*B, and *rpo*B genes was performed. All *P. ananatis* strains isolated in Poland, grouped into one cluster supported with high bootstrap value. Pathogenicity tests performed on four varieties of wheat plants have identified *P. ananatis* strains as a causal agent of wheat disease. To our knowledge, this is the first report of *P. ananatis* affecting wheat plants.

## 1. Introduction

Wheat (*Triticum aestivum*) is one of the oldest and most widespread crop species worldwide. Its world production, in 2018, was 734 million tons (http://www.fao.org/faostat/en/#data/QC/visualize), which constitutes more than 35% of the world’s food production. The micronutrients provided by wheat-based foods are essential for normal development of humans from childhood to adulthood [1]. In Poland, its acreage is estimated at 2.4 million ha and the crop is estimated at 10.9 millions of tons, which makes Poland one of the European leaders of wheat production [2]. Moreover, wheat acreage and importance are continuously increasing worldwide (https://www.statista.com/statistics/267268/production-of-wheat-worldwide-since-1990/). To optimize crop production, wheat fields are monitored for the presence of various pests, including cereal leaf beetle (CLB, *Oulema melanopus* L., Coleoptera: Chrysomelidae). This insect pest of cereal crops is particularly harmful to wheat, oat and barley. The cereal leaf beetle has a single generation per year [3]. The insect’s feeding causes the removal of chlorophyll; hence, fields may appear as though they have been damaged by frost [4]. Both beetles and larvae cause damage to plants but larvae are considered as the most damaging stage. Their feeding, especially on the flag and the subflag leaves, has a significant impact on the quality and quantity of the obtained crop. Further, CLB feeding damages the plant’s tissue leaving the open gate for microbial infection. Noteworthy, during insect feeding, the plant tissue has direct contact with its oral secretion, that might contain various biological components like viruses, bacteria, or fungi, that might be plant-pathogenic [5]. In the field conditions, regardless of insect’s presence, wheat plants might be affected by several bacterial pathogens like *Xanthomonas translucens* pv. *translucens* causing bacterial chaff of wheat [6], *Pseudomonas syringae* pv. *atrofaciens* causing a basal glume rot [7], *Bacillus megaterium* pv. *cerealis* causing white blotch [8], *Pseudomonas cichorii* causing stem melanosis [9], *Erwinia rhapontici* causing pink grain of wheat [10], and other diseases caused by *Pseudomonas syringae* pv. *syringae* van Hall [11], *Clavibacter michiganensis* subsp. *tessellarius* [12], and *Rathayibacter tritici* [13]. Moreover, *Pantoea ananatis* is known to be a causal agent of disease of other monocotyledonous crops like maize [14], sudangrass [15], or rice [16]. 

In this study, we describe our finding that the *P. ananatis* is associated with wheat plants displaying simultaneously the symptoms resembling bacterial disease and CLB feeding. It was shown that *P. ananatis* strains obtained from symptomatic wheat plants and CLB can elicit the disease symptoms in the healthy mechanically wounded plants. We suggest that the CLB might be a reservoir of *P. ananatis*.

## 2. Results

### 2.1. Field Observations

**Figure 1:**
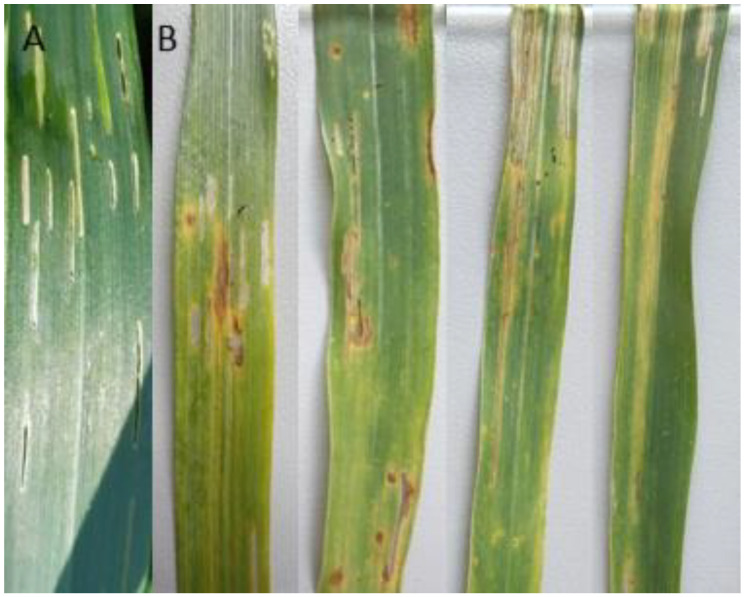
The disease symptoms observed on the wheat leaves from Winna Góra fields, with visible cereal leaf beetle (CLB) feeding wounds **(A)**. Visible CLB feeding wounds and brownish lesions with clear margins and yellow halo suggesting bacterial infection **(B).**

The plant samples expressing three types of symptoms during the routine wheat monitoring were analyzed: wheat leaves damaged only by CLB feeding (group 1), wheat leaves showing only the dark brown lesions with yellow halo suggesting the possible bacterial disease development (group 2), and leaves showing simultaneously the symptoms of CLB feeding, and lesions with yellow halo suggesting the possible bacterial disease development (group 3). The severity of CLB feeding symptoms observed during the routine monitoring was visually assessed at ~60%. Of the symptomatic plants, ~10% displayed both the CLB feeding and the lesions with a yellow halo. The observed symptoms of the third group, caused by the *P. ananatis* wheat pathogenic strains, are presented in Figure 1.

### 2.2. Identificaiton and Characteristics of Bacterial Strains

The presence of the bacterial pathogens was confirmed by observing the bacterial streaming from the original lesions (Figure 1B). After incubation, the agar surface was dominated by yellow colored, entirely smooth and glistening bacterial colonies, which morphologically corresponded to the *P. ananatis* description. 

In total, 32 pure culture strains were obtained from plant materials and identified using the Biolog Gen III system (Table 1). They belonged to: *Pseudomonas fluorescens* (3 strains), *Bacillus licheniformis* (6), *Macrococcus bunensis* (3), *Pantoea agglomerans* (11), *Pantoea ananatis* (4), *Bacillus pseudomycoides* (1), *Pseudomonas flavescens* (2), and *Pseudomonas synthaxa* (2). 

Based on the pathogenicity tests it was indicated that only four identified strains of *P. ananatis* were pathogenic, while other identified strains were non-pathogenic.

Moreover, the mentioned four strains were detected only in the group 3, where the plant samples displayed simultaneously the symptoms of CLB feeding and lesions with yellow halo suggesting the possible bacterial disease development. 

In total 18 strains were obtained from the insect’s oral secretion and identified using the Biolog Gen III system: *Acinetobacter johnstonii* (2 strains), *Enterobacter aerogenes* (1), *P. agglomerans* (3), *P. ananatis* (4), *Serratia liquefaciens* (1), *S. marcescens* (2), and 5 strains without any reference in the Biolog’s Gen III database (No ID) (Table 2). Of all tested strains, only the strains of *P. ananatis* turned out to be plant-pathogenic (Table 2). Considering this result and the fact that *P. ananatis* is known as a broad-host plant pathogen, we focused our attention on this bacterial species and its potential connection to CLB and symptom induction on plants.

The identification of *P. ananatis* strains from wheat (Ta024, Ta027, Ta030, and Ta046) and from CLB oral secretions (OUC1, OUD2, OUF2, and OUG1) was confirmed by sequencing of the partial *gyr*B genes and 16S rDNA. The comparison of nucleotide sequences obtained for the *P. ananatis* strains in this study with those deposited in the GenBank database showed the high identity level, 99-100% for both 16S rRNA and *gyr*B genes (Supplementary Tables S1 and S2). 

**Figure 2:**
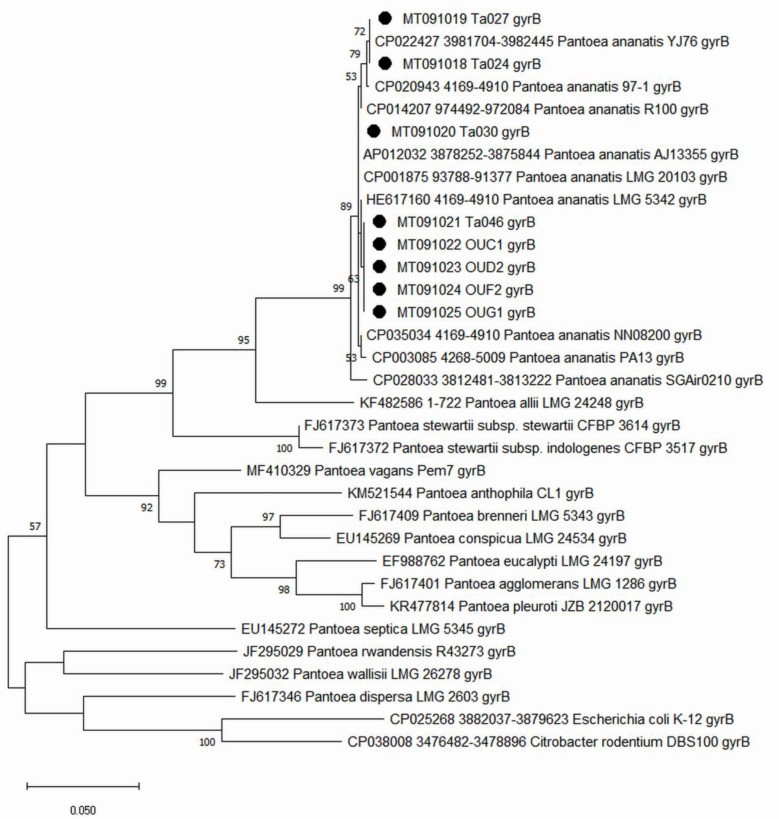
Maximum likelihood dendrogram of the partial, GenBank derived, *gyr*B gene nucleotide sequences of *Pantoea ananatis* and closely related species. Bootstrap values for phylogenetic comparisons were based on 1000 pseudoreplicates. The Polish strains are marked with black circles (●). *Escherichia coli* and *Citrobacter rodentium* strains were used as an outgroup. The bootstrap values lower than 50 were hidden.

Maximum Likelihood dendrogram based on *gyr*B gene and 16S rRNA region, both comprising eight identified *P. ananatis* strains revealed that these strains grouped together with other GenBank deposited *P. ananatis* strains (Figure 2 and Supplementary Figure S1).

**Figure 3:**
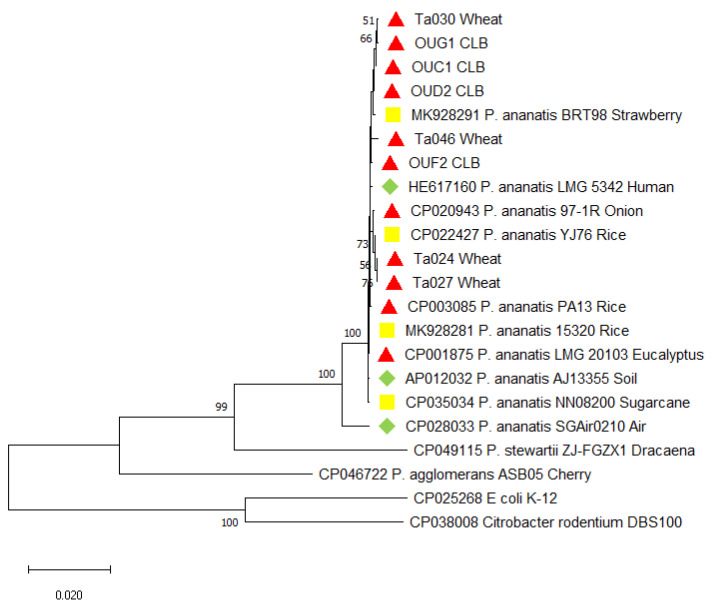
Maximum likelihood tree based on the concatenated nucleotide sequences of *atp*D, *fus*A, *gyr*B, *rpl*B, and *rpo*B of 18 *Pantoea ananatis* strains. Bootstrap values are based on 1,000 replications. Values lower than 50 were hidden. The Polish strains obtained from wheat leaves are Ta024, Ta027, Ta030, Ta046; obtained from CLB’s oral secretion: OUC1, OUD2, OUF2, OUG1. The plant-pathogenic strains of *P. ananatis* are marked with red triangle (▲); endophytic strains with yellow square (■); and environmental strains with green diamond (♦). The *Pantoea stewartii* subsp. *stewartii* and *Pantoea agglomerans* sequences were used as examples of species closely related to *Pantoea ananatis*. *Escherichia coli* and *Citrobacter rodentium* were used as an outgroup. Scale bar represents the phylogenetic distance.

The MLSA analysis (Figure 3) performed on five housekeeping genes of *P. ananatis* showed that all analyzed *P. ananatis* strains grouped into one cluster supported with a high bootstrap value and comprising two subclusters. The first subcluster contains the eight wheat-pathogenic *P. ananatis* strains described in this study, as well as other plant pathogens (PA13, 97-1) [17,18] and endophytic strains of rice, sugarcane and strawberry plants (YJ76, NN08200, BRT98, and 15320) [19,20], as well as environmental strains obtained from various ecological niches like soil (AJ13355) [21] and human wound (LMG5342) [22]. The second subcluster is formed by only one *P. ananatis* strain (SGAir0210) obtained from an outdoor air [23].

### 2.3. Pathogenicity Assessment of Strains

**Figure 4:**
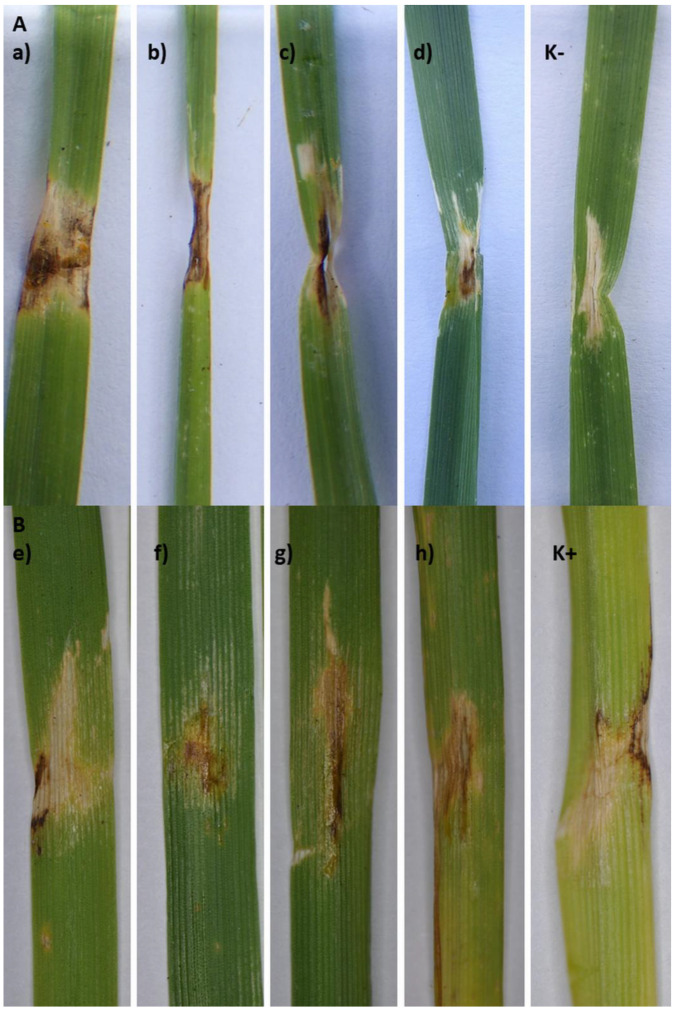
Wheat leaves with symptoms caused by **A**—Bacterial strains obtained from wheat leaves: a) Ta024 on *var*. Banderola; b) Ta027-*var*. Ostroga; c) Ta030-*var*. Arabella, d) Ta046-*var*. Arabella; **B**—Bacterial strains obtained from CLB oral secretion: e) OUF2-*var*. Banderola, f) OUD2-*var*. Banderola; g) OUC1-*var*. Arkadia; h) OUG1-*var*. Ostroga; K− negative control (water); K+ positive control *P. ananatis* ATCC 33244.

The eight strains identified as *P. ananatis*: Ta024, Ta027, Ta030, Ta046, OUC1, OUD2, OUF2, and OUG1 caused the disease symptoms on four tested wheat varieties (Arabella, Arkadia, Banderola, and Ostroga). The symptoms caused in the greenhouse resembled those observed in the field - brownish lesions with clear margins and yellow halo (Figure 1B, and Figure 4, Table 1 and Table 2). The re-isolation confirmed the presence of the *P. ananatis* strains, which has fulfilled the Koch’s postulates.

## 3. Discussion

In this study, we have investigated the symptomatic wheat leaves and the CLB imago`s oral secretions against the presence of plant pathogenic bacteria. Wheat leaves with three groups of symptoms were analyzed: 1) leaves damaged by CLB feeding, 2) leaves showing only the dark brown lesions with yellow halo suggesting the possible bacterial disease development, and 3) leaves showing simultaneously the symptoms of CLB feeding and lesions with yellow halo suggesting the possible bacterial disease development. The third group of symptoms was assessed as a significant part of field observations. All strains obtained in the study were subjected to the phytophatogenicity tests in the greenhouse. Only the *P. ananatis* strains caused the disease symptoms on wheat leaves. Moreover, those strains were recorded only on plants with the third group of symptoms and in the CLB`s oral secretions. This finding is important for three reasons. Firstly, this is the first report of *P. ananatis* on wheat plants as a host. Secondly, we showed that *P. ananatis* is able to cause the disease on a wheat plants (Figure 4). Lastly, the confirmed occurrence of *P. ananatis* strains in the CLB`s oral secretions suggests that CLB might be *P. ananatis* reservoir. Insects are known to be vectors of *P. ananatis* [24,25,26,27,28]. During insect`s feeding the plant tissue has a direct contact with its oral secretion, which might contain various biological components like viruses, bacteria, fungi, or nematodes [5]. In nature, plant injuries greatly favor a bacterial infection and the disease development [29]. That is why the wounds resulted from insects feeding on leaf plants are considered as an important entrance gate for plant pathogenic bacteria [30]. In our study, the presence of *P. ananatis* was revealed only in CLB damaged plants also displaying the yellow halo around the damaged by CLB leaf tissue. It was reported that phytopathogenic bacteria can exploit insects as their primary hosts. This process is evolutionary effective and stable in case of overlapping ecological niches when both bacterial pathogen and insect pest depend on the plant as their primary source of nutrition. Those conditions are necessary for insects to contact and encounter or ingest phytopathogenic bacteria [30]. 

Considering the ability of *P. ananatis* for colonizing of various ecological niches, and its confirmed presence in the gut microbiome of many insect’s species, e.g., *Diabrotica virgifera* [24], tobacco thrips [25], onion thrips [25], cotton fleahoppers (*Pseudatomoscelis seriatus*) [31], mulberry pyralid (*Glyphodes pyloalis*) [32], ticks, lice, and fleas [33], we suggest that there might be a connection between the CLB feeding and *P. ananatis* occurrence in wheat. Moreover, the CLB-obtained *P. ananatis* strains cause the disease symptoms on wheat. In such a case, the *P. ananatis* might pose a considerable threat not only to wheat crops but for all crops affected by insects with *P. ananatis* as a component of the gut microflora, which needs further investigation. All described features of *P. ananatis* suggest that this species might pose a great danger for the crops. This bacterium is ubiquitous, metabolically versatile, easily colonizes new and unusual ecological niches. Most importantly, however, at the current state of knowledge, it is impossible to distinguish the pathogenic strains of *P. ananatis* from non-pathogenic ones in other way than a pathogenicity test. The MLSA analysis is not resolutive enough to genetically discriminate the *P. ananatis* strains (Figure 3). Our results in this matter are congruent with the results obtained for onion-pathogenic *P. ananatis* strains [17]. Extensive genetic diversification of *P. ananatis* was described as a result of pan-genome analyses which revealed the significant role of an extensive accessory genome made up largely by a mobilome of plasmids, integrated prophages, integrative and conjugative elements, and insertion elements [34]

In conclusion, we state that the *P. ananatis* might pose a considerable threat to wheat crops on the whole area of CLB distribution which includes the whole Europe [35], Middle-East, North Asia, Africa, and eastern part of North America (https://gd.eppo.int/taxon/LEMAME/distribution). To our knowledge, this is the first report of *P. ananatis* affecting wheat plants.

## 4. Materials and Methods 

### 4.1. Plant and Insect Material 

Wheat plant samples (var. Arabella) were collected in Winna Góra (GPS: 16.801944, 50.850833) in the Greater Poland region of Poland in July 2018 and 2019. Harvested plant material was divided into three groups: 1) leaves damaged by CLB adult feeding, 2) leaves showing only the dark brown lesions with yellow halo suggesting the possible bacterial disease development, and 3) the leaves showing simultaneously the symptoms of CLB feeding and lesions with yellow halo suggesting the possible bacterial disease development (Figure 1). Each type of symptoms was investigated separately and each type of symptoms was equally represented in the harvested plant samples. In total, six plant samples were tested per season. Each sample consisted of 10 symptomatic leaves (Table 1).

The adult specimens of CLB were collected from the same wheat fields from which the plant samples were harvested. In total, six CLB samples were investigated. Each sample consisted of ten, pulled CLB specimens. Three samples were investigated each season (Table 2). 

### 4.2. Bacteria Isolation from Plants

The wheat leaves were surface sterilized by rinsing in 70% ethanol for 3 min, three times in sterile distilled water to wash the ethanol residues. The presence of bacterial streaming from the lesions was tested under the optical microscope. Next, leaves were homogenized in sterile physiological saline (0.9% NaCl). The homogenate was diluted (1:10 v/v) with sterile 1 × phosphate-buffered saline pH 7.0, and spread on TSA (Tryptic Soy Agar) medium (Sigma Aldrich Ltd., Darmstadt, Germany) to detect the potential bacterial causal agent. After incubation (27 °C for 48 h), all observed morphotypes were substreaked to new TSA plates and pure culture of each morphotype was obtained. A Gram staining and visual inspection under the optical microscope were performed to confirm the purity of each obtained culture (strain). 

### 4.3. Bacteria Isolation from Insects

The adult specimens of CLB were collected using a swiping net, then sacrificed and sterilized by rinsing in the pure ethanol (96%) for 2 min. Next, the insects were washed 3 times in 70% ethanol for the disinfection purposes and 3 times in sterile, distilled water. Finally, using the sterile forceps, each tested specimen was gently squeezed to cause the leakage of oral secretion. A total of six insect samples were tested. Each sample consisted a CLB oral secretions pulled of 10 insect specimens. The samples were placed directly and spread on the TSA medium, then incubated in 27 °C for 48 h. Obtained morphotypes were substreaked as described above. 

### 4.4. Bacterial Identification, DNA Extraction, and Sequence Analysis

Bacterial strains were identified biochemically using Biolog Gen III system (database v 2.8) (BIOLOG Inc. Hayward, CA, United States of America). The identification of plant-pathogenic strains was confirmed with a molecular method. The genomic DNA was isolated using a standard CTAB protocol [36]. For identification purposes, ~1.5 kb- and ~0.9 kb-long partial sequences of *gyr*B gene and 16S rDNA, respectively, were amplified using TD-PCR protocol [37] with degenerated *gyr*B primers designed for enterobacterial species 01-F/02-R and universal 16S rRNA bacterial primers 16S01 [38]/16S-REV [39]. The PCR products were cleaned and subjected to sequencing (Genomed S.A., Warsaw, Poland). The obtained nucleotide sequences were aligned, and the consensus sequences were verified using the BLAST tool (https://blast.ncbi.nlm.nih.gov/Blast.cgi) and submitted to the GenBank database (Figure 2, Table 1 and Table 2). The tested sequences were aligned with the sequences of *P. ananatis* strains, and with the reference strains of other *Pantoea* species deposited in the GenBank database (Supplementary Tables S1 and S2). 

### 4.5. Phylogenetic Analysis of the Tested Pantoea Ananatis Strains

With sequenced *gyr*B and 16S rRNA regions, two phylogenetic trees were computed using maximum-likelihood (ML) analysis, based on the Tamura-Nei model of MEGA X (bootstrap = 1000 repetitions) (Figure 2 and Supplementary Figure S1). The tested sequences were aligned with the sequences of 11 *P. ananatis* strains, for which a whole-genome were published and with the reference strains of other *Pantoea* species deposited in the GenBank database to investigate the phylogenetic relationships between the studied strains. For the multi-locus sequence analysis (MLSA) approach, the following partial sequences of genes were used: *atp*D = 657 bp, *gyr*B = 742 bp [40], *fus*A = 639 bp, *rpl*B = 343 bp, and *rpo*B = 596 bp [17]. Obtained partial gene sequences were aligned, trimmed, and translated (BioEdit v.7.0.5.3) [41]. For each strain the peptide coding sequences were aligned to obtain the consensus nucleotide sequences that were used to perform maximum-likelihood (ML) analysis, resulting in generating phylogenetic trees based on the Tamura–Nei model of MEGA X (bootstrap = 1000 repetitions). The GenBank derived nucleotide sequences of *Pantoea stewartii* and *Pantoea agglomerans* along with the sequences of *E. coli* K-12 and *C. rodentium* DBS100 strains were used as an outgroup as described [40] (Figure 3). 

### 4.6. Assessing Pathogenicity of Bacterial Strains

All identified bacterial strains were subjected for pathogenicity tests, using four wheat varieties (Arabella—spring wheat, Arkadia, Banderola, and Ostroga—winter wheat) at the five-leaf stage, to verify Koch’s postulates. The wheat plants were grown from seeds in the greenhouse. Just prior the inoculation the leaves were wounded with a sterile needle imitating the damage caused by the CLB. Next, the bacterial water suspension (10^8^ cfu/mL) was inoculated on the wounded area (2 mL). Each bacterial strain was tested on each wheat variety in five biological replications. Water was used as a negative control, and *P. ananatis* ATCC 33244 reference strain (DSM 17873) was used as a positive control. After inoculation, the plants were incubated in the humid chamber at 22 °C for 72 h, and kept in a greenhouse (20–25 °C, humidity 50–80%) for 14 days. The symptoms development was assessed and all symptomatic plants were subjected to the re-isolation, to confirm the presence of inoculated bacteria (Koch`s postulate), as described above. In the field, the severity of CLB feeding symptoms observed during the routine monitoring was visually assessed (Figure 1B and Figure 4).

## Figures and Tables

**Table 1:** Bacterial strains obtained from the collected wheat plant samples var. Arabella.

No	Strain Tested	Symptoms Group ^1^/Season	Biolog Gen III Identification Result	Wheat Pathogenic (Yes/No)	16S rDNA Sequencing Result (GenBank Accession Number)	*gyr*B Sequencing Result (GenBank Accession Number)
Season 2018						
1	Ta015	1/2018	*Bacillus licheniformis*	No	nt	nt
2	Ta016	1/2018	*Bacillus licheniformis*	No	nt	nt
3	Ta017	1/2018	*Pantoea agglomerans*	No	nt	nt
4	Ta018	1/2018	*Bacillus licheniformis*	No	nt	nt
5	Ta019	1/2018	*Macrococcus brunensis*	No	nt	nt
6	Ta020	1/2018	*Macrococcus brunensis*	No	nt	nt
7	Ta021	1/2018	*Pantoea agglomerans*	No	nt	nt
8	Ta022	1/2018	*Pantoea agglomerans*	No	nt	nt
9	Ta023	2/2018	*Bacillus licheniformis*	No	nt	nt
10	Ta024	3/2018	*Pantoea ananatis*	Yes	MH973236	MT091018
11	Ta025	3/2018	*Bacillus licheniformis*	No	nt	nt
12	Ta026	3/2018	*Pseudomonas fluorescens*	No	nt	nt
13	Ta027	3/2018	*Pantoea ananatis*	Yes	MH973237	MT091019
14	Ta028	2/2018	*Pantoea agglomerans*	No	nt	nt
15	Ta029	3/2018	*Pseudomonas fluorescens*	No	nt	nt
16	Ta030	3/2018	*Pantoea ananatis*	Yes	MH973238	MT091020
17	Ta031	2/2018	*Bacillus licheniformis*	No	nt	nt
18	Ta032	2/2018	*Macrococcus brunensis*	No	nt	nt
19	Ta033	2/2018	*Bacillus pseudomycoides*	No	nt	nt
20	Ta034	2/2018	*Pseudomonas flavescens*	No	nt	nt
21	Ta035	2/2018	*Pseudomonas flavescens*	No	nt	nt
22	Ta036	2/2018	*Pantoea agglomerans*	No	nt	nt
Season 2019						
23	Ta037	2/2019	*Pantoea agglomerans*	No	nt	nt
24	Ta038	2/2019	*Pantoea agglomerans*	No	nt	nt
25	Ta039	2/2019	*Pseudomonas synthaxa*	No	nt	nt
26	Ta040	2/2019	*Pseudomonas synthaxa*	No	nt	nt
27	Ta041	1/2019	*Pantoea agglomerans*	No	nt	nt
28	Ta042	1/2019	*Pantoea agglomerans*	No	nt	nt
29	Ta043	1/2019	*Pantoea agglomerans*	No	nt	nt
30	Ta044	1/2019	*Pantoea agglomerans*	No	nt	nt
31	Ta045	3/2019	*Pseudomonas fluorescens*	No	nt	nt
32	Ta046	3/2019	*Pantoea ananatis*	Yes	MH973239	MT091021

^1^ The symptoms observed on analyzed plants: 1) leaves showing only the symptoms of CLB feeding, 2) leaves showing only the dark brown lesions with a yellow halo, 3) the leaves showing simultaneously the symptoms of CLB feeding and lesions with yellow halo; nt—not tested.

**Table 2:** Bacterial strains obtained from the oral secretion of the adult specimens of *Oulema melanopus* collected on wheat plants *var*. Arabella; nt—not tested; No ID—no reference in the Biolog Gen III database (v. 2.8.0).

No	Strain Tested	Sample/Season	Biolog Gen III identification Result	Wheat Pathogenic (Yes/No)	16S rDNA Sequencing Result (GenBank Accession Number)	*gyr*B Sequencing Result (GenBank Accession Number)
Season 2018						
1	OUC1	1/2018	*Pantoea ananatis*	Yes	MT234395	MT091022
2	OUC2	1/2018	*Pantoea agglomerans*	No	nt	nt
3	OUC3	1/2018	*Serratia liquefaciens*	No	nt	nt
4	OUD1	2/2018	*Pantoea agglomerans*	No	nt	nt
5	OUD2	2/2018	*Pantoea ananatis*	Yes	MT234396	MT091023
6	OUD3	2/2018	*Serratia marcescens*	No	nt	nt
7	OUE1	3/2018	No ID	No	nt	nt
8	OUE2	3/2018	No ID	No	nt	nt
9	OUE3	3/2018	No ID	No	nt	nt
Season 2019						
10	OUF1	1/2019	*Pantoea agglomerans*	No	nt	nt
11	OUF2	1/2019	*Pantoea ananatis*	Yes	MT234397	MT091024
12	OUF3	1/2019	*Acinetobacter johnstonii*	No	nt	nt
13	OUG1	2/2019	*Pantoea ananatis*	Yes	MT234398	MT091025
14	OUG2	2/2019	*Serratia marcescens*	No	nt	nt
15	OUG3	2/2019	*Enterobacter aerogenes*	No	nt	nt
16	OUH1	3/2019	*Acinetobacter johnstonii*	No	nt	nt
17	OUH2	3/2019	No ID	No	nt	nt
18	OUH3	3/2019	No ID	No	nt	nt

## Supplementary Materials

The following are available online at https://www.mdpi.com/2076-0817/9/12/1079/s1. Table S1. BLAST sequence identity (%) of the *gyr*B sequences of the tested strains with other *Pantoea ananatis* sequences derived from the GenBank database. Table S2. BLAST sequence identity (%) of the 16S rRNA sequences of the identified strains with other *Pantoea ananatis* sequences derived from GenBank database. Figure S1. Maximum likelihood dendrogram of the partial, GenBank derived a nucleotide sequence of 16S rRNA gene of *Pantoea ananatis* and closely related species. Bootstrap values for phylogenetic comparisons were based on 1000 pseudoreplicates. The Polish strains are marked with black squares. *Escherichia coli* and *Citrobacter rodentium* strains were used as an outgroup. The bootstrap values lower than 50 were hidden. Scale bar represents the phylogenetic distance.
