# Supplementary material for: Pantoea ananatis, A New Bacterial Pathogen Affecting Wheat Plants (Triticum L.) in Poland
Source: Pathogens. 2020 Dec 21;9(12):1079. doi: 10.3390/pathogens9121079 (PMC7767503; doi:10.3390/pathogens9121079)
Supplement: Supplementary file 1 [file pathogens-09-01079-s001.pdf]

pathogens

MDPI

Supplementary Materials

Table S1. BLAST sequence identity (%) of the *gyr*B sequences of the tested strains with other *Pantoea ananatis* sequences derived from the GenBank database.

Tested strain code /GenBank accession number	Gene tested	Amplicon size (bp)	Identity with the BLAST best hit result (%)	The most similar GenBank derived *Pantoea ananatis* sequence (accession no)
Ta024-MT091018	*gyr*B	864	100	CP022427
Ta027-MT091019	*gyr*B	864	100	CP022427
Ta030-MT091020	*gyr*B	864	100	MK928262
Ta046-MT091021	*gyr*B	864	99	MK928262
OUC1-MT091022	*gyr*B	864	99	MK928262
OUD2-MT091023	*gyr*B	864	99	MK928262
OUF2-MT091024	*gyr*B	864	99	MK928262
OUG1-MT091025	*gyr*B	864	99	MK928262

Table S2. BLAST sequence identity (%) of the 16S rRNA sequences of the identified strains with other *Pantoea ananatis* sequences derived from GenBank database.

Tested strain code /GenBank accession number	Gene tested	Amplicon size (bp)	Identity with the BLAST best hit result (%)	The most similar GenBank derived *Pantoea ananatis* sequence (accession no)
Ta024- MH973236	16S rRNA	1494	99	CP022427
Ta027- MH973237	16S rRNA	1532	99	DQ517335
Ta030- MH973238	16S rRNA	1527	99	DQ195525
Ta046- MH973239	16S rRNA	1488	100	KT957000
OUC1-MT234395	16S rRNA	1445	100	MK415050
OUD2-MT234396	16S rRNA	1477	100	MK415050
OUF2-MT234397	16S rRNA	1489	99	MK415050
OUG1-MT234398	16S rRNA	1443	99	MK415050

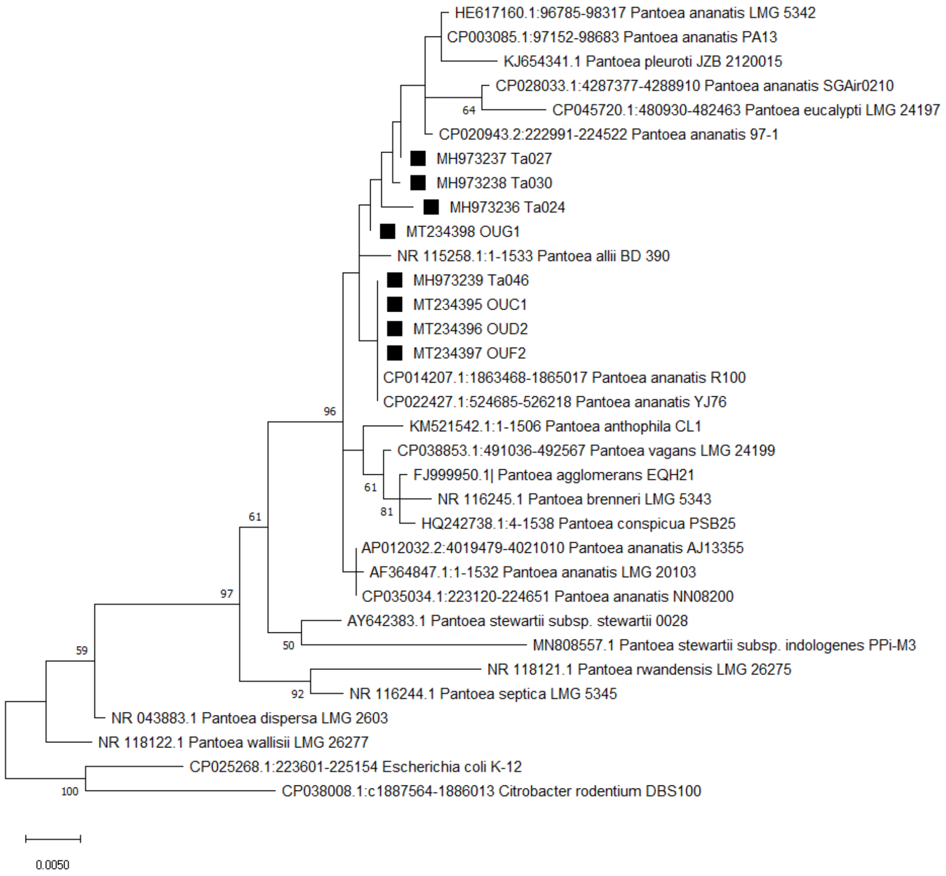

Figure S1. Maximum likelihood dendrogram of the partial, GenBank derived a nucleotide sequence of 16S rRNA gene of *Pantoea ananatis* and closely related species. Bootstrap values for phylogenetic comparisons were based on 1000 pseudoreplicates. The Polish strains are marked with black squares (■). *Escherichia coli* and *Citrobacter rodentium* strains were used as an outgroup. The bootstrap values lower than 50 were hidden. Scale bar represents the phylogenetic distance.
